# Neoadjuvant Chemotherapy for Colon Cancer

**DOI:** 10.3390/cancers12092368

**Published:** 2020-09-26

**Authors:** Marc T. Roth, Cathy Eng

**Affiliations:** Division of Hematology/Oncology, Department of Internal Medicine, Vanderbilt University Medical Center, Nashville, TN 37232, USA; cathy.eng@vumc.org

**Keywords:** colon cancer, neoadjuvant, chemotherapy, immunotherapy, microsatellite instability, MSI-H

## Abstract

**Simple Summary:**

Neoadjuvant chemotherapy is commonly used in several solid tumor malignancies, but remains understudied in the setting of locally advanced colon cancer. Advantages of this strategy extrapolated from other disease sites include early treatment of micro-metastatic disease, the ability to decrease local disease burden potentially leading to more effective resections and improved treatment tolerability. Approaches for accurate staging and safe administration of systemic treatment are being investigated in large, randomized clinical trials, but available data are either not mature enough or have not demonstrated a convincing argument for adoption into standard practice warranting further investigation.

**Abstract:**

Early stage colon cancer is typically managed with surgical resection, although not all patients experience a durable remission. Adjuvant chemotherapy with a fluoropyrimidine, with or without oxaliplatin, is commonly utilized to increase the chance of cure, but its efficacy in the neoadjuvant setting is not well established. Preoperative chemotherapy has demonstrated safety and efficacy in other gastrointestinal malignancies, but there is a paucity of data from large, prospective randomized trials, although multiple are ongoing. In this review, we will discuss the theoretical risks and benefits, logistical difficulties, and available safety and efficacy data pertaining to the use of chemotherapy in locally advanced colon cancer.

## 1. Introduction

Colon cancer accounted for nearly 1.1 million new cancer cases and was responsible for over 550,000 deaths globally in 2018 [1]. Furthermore, colorectal cancers are the third leading cause of cancer-related deaths worldwide. Over 70% of patients will present with localized or regional disease, which represents the best opportunity for cure via mesocolic excision [2]. After surgery, a surveillance plan is generally set forth to detect early recurrence, including a scheduled history and physical labs including tumor markers, imaging and endoscopic examinations. Per the National Comprehensive Cancer Network (NCCN) guidelines, adjuvant chemotherapy can be considered for stage II disease, with stronger evidence to support its use as staging increases due to depth of invasion and lymph node involvement as in stage III disease [3]. Typically, a fluoropyrimidine, with or without oxaliplatin (depending on stage and presence of high-risk features), is provided to reduce the risk of disease recurrence. Even with adjuvant chemotherapy, the risk of recurrence at five years for colorectal cancer can be over 25% [4].

Recently, there has been increasing interest in the utilization of neoadjuvant chemotherapy (NAC) in colon cancer. Although few prospective randomized clinical trials have been conducted to inform the subject, retrospective studies and small institutional trials have postulated there may be some benefit. NAC is already widely used in other gastrointestinal malignancies, including esophageal, gastric and rectal cancers [5,6]. Multiple theories exist regarding the potential advantages in locally-advanced colon cancer (LACC). First, NAC may aid in the earlier eradication of micro-metastatic disease and decrease the size/stage of the primary tumor. This, in turn, could lead to increased R0 (margin negative) resection rates. Animal models have demonstrated that surgical trauma leads to locoregional metastasis [7]; however, through cytotoxic debulking with NAC, tumor cell shedding could decrease during surgery. In addition, multiple observational studies have shown that chemotherapy is better tolerated preoperatively, leading to fewer delays [8,9].

Neoadjuvant chemotherapy is not without its risks, however. Peripheral neuropathy from oxaliplatin is a major morbidity incurred from adjuvant chemotherapy for colorectal cancer, so much so that large clinical trials have investigated the efficacy of shortening durations of treatment [10]. Moving treatment into the pre-surgical space could lead to the overtreatment of low-risk patients if they have incorrect radiographic staging [11,12]. Although NAC allows observation of disease biology and assessment of chemo-responsiveness, delaying surgery in nonresponsive tumors could lead to tumor growth, predisposing patients to obstruction and/or perforation, requiring an emergent surgery associated with high morbidity and mortality.

This article will discuss the evidence for and against the use of neoadjuvant chemotherapy in locally advanced colon cancer, with a special focus on recent randomized clinical trials and implications of molecular subclasses. A comprehensive search of the literature was conducted for all clinical trial phases involving neoadjuvant chemotherapy use in non-metastatic colon cancer. This search included PubMed, clinicaltrials.gov and a review of all major conference abstracts. Of note, rectal cancer and chemoradiation is excluded from this chapter and will be discussed elsewhere in this issue. 

## 2. Radiographic Staging

An integral component of the appropriate use of NAC in the preoperative setting in colon cancer is the ability to accurately stage patients via imaging. Traditionally, adjuvant therapy is indicated based on pathologic staging—the gold standard. However, pathologic staging becomes less useful in dictating the need for adjuvant treatment after exposure to cytotoxic chemotherapy due to expected tumor regression. Early studies utilizing computed tomography (CT) to stage LACC found that T and N staging was correctly predicted by radiologists in 60% and 62%, respectively [11]. A pilot study for the FOxTROT clinical trial (discussed below) also assessed radiologist’s staging accuracy within their protocol, finding that sensitivity and specificity for identifying T3/T4 vs. T1/T2 through tumor infiltration beyond the muscularis propria were 95% and 50%, respectively [12]. One large retrospective analysis of the National Cancer Database (NCDB) evaluated 105,569 patients with clinical and pathologic staging, finding that the correlation for the T stage was 80%, and 83% for N stage. Agreement increased with high T and N stages, suggesting that early stage disease is more difficult to accurately assess [13]. Other modalities have been evaluated for use, such as MRI [14,15] and CT colonography [16,17,18], but they have not yet replaced CT as standards within this space, in part due to cost and invasiveness. Understanding the contribution of each radiographic staging modality and when to use them may improve diagnostic accuracy further, to ensure that patients receive the appropriate treatment for their disease.

## 3. Retrospective Studies

One of the earliest groups to report the potential benefit of neoadjuvant therapy in LACC was from Arredondo et al. in 2013 [19]. Their analysis included 22 patients with stage III colon cancer who received preoperative CAPOX (capecitabine 1000 mg/m^2^ twice daily on days 1–7, oxaliplatin 85 mg/m^2^ on day 1 every other week) ×4 between 2009 and 2010. After resection, they went on to receive four additional cycles of adjuvant CAPOX. All patients experienced a radiographic response with a median of 69.5% tumor volume reduction. No progressive disease occurred during preoperative chemotherapy. At 14.4 months post-op, the actuarial overall survival (OS) was 100% and progression-free survival (PFS) was 90%. Forty-three additional patients were evaluated on the same protocol between 2009 and 2014 (infusional 5-FU was used in some cases [20]). Out of 65 total patients, the majority (93.8%) completed planned treatment and no surgeries were delayed, with a median start time of 71 days from chemotherapy to surgery. Tumor volume reduction assessed via CT was reported at 62.5%. Pathologic complete response (pCR) was seen in 4.6% of patients. Adjuvant chemotherapy was only given to 60% of patients, but five-year actuarial OS was over 95%. These findings formed the basis of ELECLA (NCT04188158); the larger, randomized phase II study of neoadjuvant CAPOX in LACC, which is ongoing.

Many of the retrospective data regarding neoadjuvant chemotherapy are derived from its use in patients with T4b disease, defined as a tumor that directly invades or adheres to adjacent organs [3]. In 2016, the option for NAC in T4b disease was added to NCCN guidelines, after which two large retrospective reviews of national databases were reported. In 2017, Dehal et al. published a retrospective analysis of 921 patients in the NCDB, who received neoadjuvant chemotherapy between 2006 and 2014 [21]. Propensity score matching was used to compare this cohort to a standard of care group treated with upfront surgery and adjuvant chemotherapy. Of note, patients treated with NAC were younger, with higher-grade histology and advanced clinical T stage, but less advanced N stage than the adjuvant group. At a median follow up of 3.6 years, 3-year OS was improved in the T4b neoadjuvant cohort at 74%, compared to 66% after adjuvant chemotherapy (Hazard ratio (HR) 0.7, 95% CI 0.56–0.87; *p* = 0.0002). This comparison remained statistically significant after propensity score matching. No survival difference was noted in the T3 or T4a cohorts.

A similar study was reported in 2019, using data from the Netherlands Cancer Registry [22]. Propensity score matching was used to evaluate 149 patients with clinical T4 LACC treated with neoadjuvant chemotherapy. In contrast to the NCDB study, R0 resection was achieved in only 77% of those treated with NAC, vs. 86% of those after adjuvant chemotherapy (*p* = 0.037). Five-year OS did not vary between the two groups (67% vs. 65%, *p* = 0.87). Still, no difference in surgical complications was found between the two groups.

## 4. Prospective Single-Arm Studies

Multiple prospective, single-arm studies have been conducted to verify feasibility of the NAC approach in LACC. Jakobsen et al. enrolled 77 patients with high risk T3/T4 colon cancer and assigned them to receive three cycles of CAPOX (capecitabine 2000 mg/m^2^ daily on days 1–14 and oxaliplatin 130 mg/m^2^ on day 1 every 3 weeks) if they harbored a KRAS, BRAF or PIK3CA mutation, or if their mutational status was unknown [23]. Wildtype patients received CAPOX plus panitumumab. Based on pathologic response, if patients normally would have received adjuvant chemotherapy, they continued to receive CAPOX ×5 cycles without panitumumab. If they were converted to a lower stage, not meeting adjuvant chemotherapy criteria, they were observed. The primary endpoint was the rate of conversion from a higher clinical stage to a lower pathologic stage no longer requiring adjuvant chemotherapy. Conversion rate in the wildtype group was 42% compared to 51% in patients with a mutation, with three patients experiencing a complete response. The 3-year DFS was 94% in the converted group, versus 63% who did not convert (*p* = 0.0005).

Shortly thereafter, a similar design was employed by Liu et al. in a single-arm phase II trial evaluating neoadjuvant CAPOX for patients with LACC [24]. Forty-seven patients were enrolled, with 42 of them completing two to four cycles of NAC (depending on response) prior to resection, followed by adjuvant chemotherapy to include eight cycles in total. The total clinical response rate was 70.7%, including one pCR and a partial response (PR) rate of 68.3%. Of note, emergent operations were observed in three patients who experienced perforation or obstruction, but perioperative morbidity and mortality were low.

After the efficacy of triplet therapy with a fluoropyrimidine, irinotecan and oxaliplatin was established in metastatic colorectal cancer and a feasibility study was conducted in localized disease in the neoadjuvant setting [25]. Twenty-three patients with stage IIIB colon cancer received four cycles of FOLFOXIRI, followed by resection and an additional six cycles of either FOLFOXIRI or CAPOX. Tumor volume reduction occurred in 91.3% of patients (including one pCR), with 56.6% experiencing grade 3–4 toxicities, although no severe complications from surgery were observed. Of note, one patient experienced a delay in surgery due to sustained bone marrow suppression and two progressed during neoadjuvant treatment. Only 52.2% of patients completed all four cycles of neoadjuvant chemotherapy, most commonly due to toxicity. At a median follow up time of 28 months, the 2-year OS rate was 95.7%, with a 2-year recurrence rate of 26.1%.

## 5. Prospective Randomized Trials

Very few prospective randomized trials utilizing neoadjuvant chemotherapy in LACC have been conducted. In 2015, a group from France published their phase II trial protocol for NAC [26]. High risk T3, T4 and/or N2 LACC patients were randomized to receive either immediate resection followed by adjuvant FOLFOX (oxaliplatin 85 mg/m^2^, folinic acid 400 mg/m^2^, 5-FU 400 mg/m^2^ bolus followed by 2400 mg/m^2^ continuous infusion over 46 h), versus four cycles of neoadjuvant FOLFOX (plus cetuximab if RAS wildtype), followed by resection and eight additional cycles of FOLFOX to complete adjuvant treatment. The primary endpoint was histological tumor regression grade (TRG), which considers the presence of viable tumor cells and fibrosis. The accrual goal was 165 patients. After the interim analysis, the data monitoring committee recommended stopping the cetuximab arm due to lack of prespecified efficacy by TRG and a severe postoperative morbidity rate of 15% in this arm. Enrollment continued into the two other arms and the results of the trial were published earlier this year [27]. Ultimately, 104 patients were included in the primary endpoint analysis. Significant pathologic regression was seen in 44% of those receiving preoperative chemotherapy versus 8% in the control arm, yet the primary endpoint in TRG was not met. In the control arm, nearly 33% of patients who were radiographically staged as high risk T3, T4 or N2 LACC were found to have low-risk stage II colon cancer and could have been overtreated (based on the current standard of care) due to clinical overstaging.

The FOxTROT phase III clinical trial is the largest reported randomized trial to date, investigating neoadjuvant chemotherapy in LACC. The primary outcome was the relapse or persistent disease rate after two years following resection. The trial was divided into a phase II lead-in to evaluate feasibility and safety [28], followed by a larger phase III period. Secondary outcomes included complete resection rate, perioperative safety, downstaging and tumor regression. The pilot study was also utilized to verify the accuracy of CT staging of high-risk colon cancers [12]. Radiologists involved in the study attended workshops in order to standardize the interpretation of good, intermediate and poor prognosis tumors. Criteria defined as poor prognoses were T3 and tumor extension ≥ 5 mm beyond the muscularis propria or T4 disease (these criteria were largely used by the trials previously mentioned). Preoperative staging was then compared to surgical pathology, identifying only 7% of patients who were definitively overstaged and would have received chemotherapy unnecessarily. 

Patients with radiologically-staged, high-risk T3–T4, N0–2 colon cancer were randomized to either (A) three cycles of FOLFOX, followed by surgery and then nine additional cycles after surgical resection, or (B) surgical resection, followed by twelve cycles of adjuvant FOLFOX. Randomization occurred in a 2:1 fashion (A:B) and 1053 patients were ultimately enrolled [29]. If patients were KRAS wildtype (*n* = 279), they were further randomized to receive panitumumab, which was to take place within six weeks of starting NAC. Two dealer’s choices were included, allowing investigators to limit total chemotherapy duration to twelve weeks (instead of 24 weeks) in older or intermediate-risk patients (T3 or < 5mm extramural extension), or utilize capecitabine instead of infusional 5-FU (excluding those receiving panitumumab). The study was conducted across 85 centers in the United Kingdom, Denmark and Sweden between 2008 and 2016.

In the initial update at ASCO 2019, the intervention was reported to be safe and well-tolerated [29]. A 4% (*n* = 25) pCR rate was reported after NAC, and perioperative morbidity was not significantly increased. The primary endpoint of 2-year failure rate was not statistically significant between the NAC and adjuvant chemotherapy arms (14% vs. 18%, HR = 0.77; *p* = 0.11). A planned interim report was provided at ASCO 2020 [30]. Neoadjuvant chemotherapy was delivered on schedule in 85% of patients, with only one delay in surgery due to neutropenia. Otherwise, there were no significant differences in post-operative morbidity or length of hospital stays between the two cohorts. Relapse or persistent disease after two years showed a trend toward improvement at 15.6% for NAC vs. 19.5% for adjuvant chemotherapy, but did not meet statistical significance (HR = 0.76, 95% confidence interval (CI 0.56–1.02; *p* = 0.07). Patients in the experimental group had significantly down-staged tumors (*p* < 0.001 for pT and pN) and fewer incomplete resections (5% vs. 10%, *p* = 0.001). In a subgroup analysis, tumor characteristics which appeared to benefit from NAC included left-sided primary tumor location (OR 0.58, 0.38–0.91) and T4 lesions (OR 0.59, 0.35–1.00). Furthermore, the addition of EGFR inhibition did not seem to affect the primary outcome.

Additional analyses were conducted comparing mismatch repair (MMR) status assessed by immunohistochemistry (IHC) in response to neoadjuvant chemotherapy. MMR status was obtained in 86.8% of patients, with 20.2% being MMR-deficient (dMMR) and 79.8% being MMR-proficient (pMMR), or unknown. As expected, based on the known behavior of dMMR tumors, a lower rate of tumor regression with chemotherapy compared to pMMR was seen (OR = 0.37, 0.22–0.61, *p* < 0.0001). Similarly, reductions in recurrence at two years were primarily seen in pMMR tumors (RR = 0.72, 0.52–1.00, *p* = 0.05), compared with dMMR tumors (RR = 0.94, 0.43–2.07, *p* = 0.9). These data, and those from other clinical trials, suggest that dMMR tumors should be treated differently than pMMR in nonmetastatic colon cancer. Studies are ongoing and discussed below.

The largest randomized phase III trial utilizing neoadjuvant CAPOX is currently recruiting in China [31]. Goal accrual is 1370 patients with a primary outcome of 3-year DFS. Objective response rate, R0 resection rate, OS and safety will be secondary endpoints. Of note, this trial will take into account MMR/MSI status when screening. Other ongoing clinical trials are listed in Table 1.

## 6. Individualized Therapy

Immune checkpoint inhibition (ICPI) has shown efficacy in metastatic colorectal cancer and was recently approved in the first-line setting for microsatellite instability “high” (MSI-H) patients. Naturally, interest in ICPI use in the neoadjuvant space in colon cancer has grown after successes in other malignancies [32,33,34]. In the exploratory NICHE study, investigators proposed utilizing ICPI in both dMMR and pMMR LACC [35]. They postulated that there may be greater efficacy in early stage pMMR colon cancer over late stage due to the higher degree of T-cell infiltration in the former [36]. Within the pMMR group, celecoxib was also added, based on preclinical evidence that it may be synergistic with ICPI and increase tumor-promoting inflammation [37]. Patients received dual ICPI with ipilimumab (day 1) and nivolumab (days 1 and 15), followed by surgery to occur within six weeks of study consent. The primary objective was safety and feasibility in this phase II study. Out of 21 dMMR patients and 20 pMMR patients enrolled, 20 with dMMR and 15 with pMMR were included in the analysis. There were no delays in surgery and adverse events were in line with the reported side effect profiles of the therapies. Within the dMMR cohort, 100% had a pathologic response (60% had a pCR). Of the pMMR tumors, 27% experienced a pathologic response (13% pCR). Four patients (1 dMMR, 3 pMMR) went on to receive adjuvant chemotherapy after resection. At a median follow up of nine months, all dMMR patients were alive and without disease. One pMMR patient developed metastatic disease treated with palliative chemotherapy, and one died due to unrelated circumstances. This study continues patient accrual.

The PePiTA study (Preoperative chemosensitivity testing as Predictor of Treatment benefit in Adjuvant stage III colon cancer) is evaluating adjuvant therapy but utilizes a component of neoadjuvant chemotherapy in its design. In this prospective, single-arm study, LACC patients received one cycle of FOLFOX, followed by surgery and standard of care adjuvant chemotherapy. PET/CT responses before and after one cycle of preoperative chemotherapy were used to predict adjuvant outcomes measured by 3-year DFS. Metabolic response on imaging was defined as a decrease in ≥ 15% in SUVmax after one cycle of chemotherapy. In a preliminary analysis, metabolic response was reported in 204/240 patients [38]. In 218 patients who received NAC, a metabolic response was observed in 65.2% of patients. Although evaluating neoadjuvant therapy was not the primary interest of this study, results may shed light on which patients are likely to benefit from chemotherapy in this setting. 

## 7. Conclusions

Neoadjuvant chemotherapy will likely find its niche in the treatment of locally advanced colon cancer as data continue to unfold. Teasing out which populations are likely to benefit through molecular characterization and radiographic response will be necessary. Likewise, avoiding operative delays in those with a low likelihood of response to cytotoxic treatment will be imperative. The inclusion of novel approaches with immunotherapy or other targeted agents outside of traditional chemotherapy could provide significant survival advantages, including for a personalized approach. The generalizability of any of these approaches must be kept in mind, especially with a burgeoning young adult cohort that may be appropriate for treatment intensification, and an elderly patient population that is above the median age reflected in the aforementioned trials. The momentum of the field is encouraging and with continued ingenuity, neoadjuvant treatment of colon cancer has the potential to evolve into a new standard of care with improved advances in diagnostic imaging, molecular characterization, and clinical trial enrollment. 

## Figures and Tables

**Table 1:** Ongoing neoadjuvant therapy trials in localized colon cancer

Trial Number	Intervention	Population	Status	Phase	Accrual Goal	Location(s)	Primary Endpoint
NCT03125980	CAPOX ×4 → surgery → CAPOX ×4 Vs. surgery → CAPOX ×8	LACC	Recruiting	III	1370	China	3-year DFS
NCT03426904	neoadjuvant FOLFOX ×4 → surgery → adjuvant FOLFOX ×8 vs. surgery → adjuvant FOLFOX × 12	LACC	Recruiting	III	560	Korea	RFS
NCT01918527	neoadjuvant CAPOX ×3 → surgery → adjuvant CAPOX (if indicated based on pathology) vs. surgery → adjuvant CAPOX ×4–8	LACC	Recruiting	III	250	Denmark Sweden Norway	2-year DFS
NCT02972541 (NACSOC)	colonic stent → FOLFOX ×3 or CAPOX ×2 → surgery → FOLFOX ×5–9 or CAPOX ×4–6 vs. colonic stent → surgery → FOLFOX ×8–12 or CAPOX ×6–8	LACC	Recruiting		248	China	DFS
NCT04188158 (ELECLA)	neoadjuvant CAPOX ×3 → surgery → adjuvant CAPOX ×5 vs. surgery → adjuvant CAPOX ×8	LACC	Recruiting	II	238	Spain	2-year DFS
NCT01675999 (ECKINOXE)	neoadjuvant FOLFOX ×4 ± cetuximab → surgery → adjuvant FOLFOX ×8 ± cetuximab vs. surgery → adjuvant FOLFOX ×12	LACC	Recruiting	II	186	France	Tumor response by TRG
NCT03026140 (NICHE)	ipilimumab + nivolumab ± celecoxib → surgery	Stages I–III CC	Recruiting	II	60	Netherlands	Safety
NCT04231526	pembrolizumab → surgery vs. surgery	LACC	Not yet recruiting	II	46	USA	Feasibility
NCT03985891	toripalimab (anti-PD1) + FOLFOX ×6 → surgery → same ×6 vs. FOLFOX ×6 → surgery → FOLFOX ×6	LACC	Not yet recruiting	I/II	40	China	pCR rate rCR rate ORR
NCT03484195	FOLFOXIRI ×4 → surgery	LACC	Not yet recruiting	II	30	China	Tumor downstaging

Abbreviations: CAPOX = capecitabine+oxaliplatin, LACC = locally advanced colon cancer, DFS = disease free survival, FOLFOX = 5-fluorouracil+folinic acid + oxaliplatin, RFS = relapse free survival, TRG = tumor response grade, CC = colon cancer, pCR = pathologic complete response, rCR = radiographic complete response, ORR = objective response rate, FOLFOXIRI = 5-FU + folinic acid + oxaliplatin + irinotecan.
